# Supplementary material for: Enhanced Quinolone Resistance and Differential Expression of Efflux Pump nor Genes in Staphylococcus aureus Grown in Platelet Concentrates
Source: Antibiotics (Basel). 2025 Jun 21;14(7):635. doi: 10.3390/antibiotics14070635 (PMC12291658; doi:10.3390/antibiotics14070635)
Supplement: Supplementary file 1 [file antibiotics-14-00635-s001.zip › antibiotics-3687635-supplementary.pdf]

Supplementary Materials

Table S1. Minimal Inhibitory Concentration (MIC) of ciprofloxacin and norfloxacin in *S. aureus* grown in Müeller Hinton (n≥3).

Strains	Ciprofloxacin (μg/mL)	Norfloxacin (μg/mL)
ATCC 29213 (Control strain)	0.125 – 0.5	1 – 4
CBS 2016-05	0.25 – 0.5	0.5
CI/BAC/25/13/W	0.5 – 1	2 – 4
PS/BAC/169/17/W	0.5 – 1	0.5 – 2
PS/BAC/317/16/W	0.5 – 1	0.25 – 1
RN6390 "wild type"	0.125 – 0.5	0.5 – 2
RN6390Δ*norB*	0.125 – 0.5	0.25 – 0.5
RN6390Δ*mgrA*	0.125 – 0.5	0.5 – 1

Table S2. Overview of *S. aureus* isolates genome features (two TRS and RN6390)

Strain	CBS 2016-05	CI/BAC/ 25/13/W	PS/BAC/ 169/17/W	PS/BAC/ 317/16/W	RN6390
Accession number	NZ_CP070991	NZ_CP071102	NZ_CP071100	NZ_CP071104	NZ_CP090001
Origin	PCs				Laboratory
Genome size (bp)	2,766,936	2,719,347	2,753,746	2,665,983	2,740,225
Genes (total)	2,823	2715	2,739	2,609	2,717
GC content (%)	32.87	32.88	32.85	32.93	32.90
CDSs	2,741	2,629	2,658	2,392	2,635
rRNA	19	19	19	19	19
tRNA	59	60	58	58	59
ncRNA	4	4	4	4	4
Reference	[28]	[29]	[30]	[31]	[37]

Table S3. List of primers in this study (5′ ® 3′).

Primer	Sequence
**RT-qPCR**	
16S RNA (forward)	AAG TCG ATG GGC AAG ATG ATA C
16S RNA (reverse)	TCC TTC GTG AAG CTC CAT TTC
norA (forward)	GAC CAG GGA TTG GTG GAT TTA T
norA (reverse)	GGA AAC CAC TTG TCG TAG ACT T
norB (forward)	GAA TTA GGT GTA ACC TCA CTT CTT T
norB (reverse)	GTT GCA CCT GTG TAA GCT TAT
norC (forward)	TGG CAG TGG TAT CTG TTC AC
norC (reverse)	GGC GTC CCT TTG ATG AGT AA
**Allelic exchange - *norB* mutant**	
norB-A(KpnI)	ATG CGG TAC CAT TCG CAA AAT TAA CAA AAT
norB-B	CAT CTC TAT TTG CCT CCC TAT ACT TTT GA
norB-C	GGC AAA TAG AGA TGT AAT TGA GAA TTA AAT
norB-D (SacI)	TAC TTT GTT GTT TCT GTT TAG AGC TCG CAT

(A)

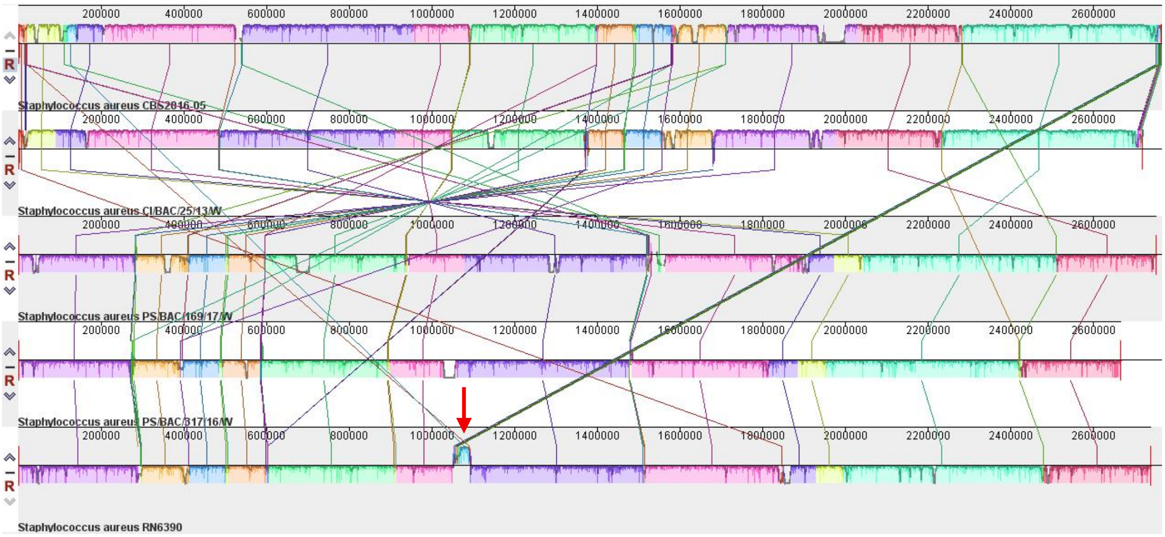

(B)

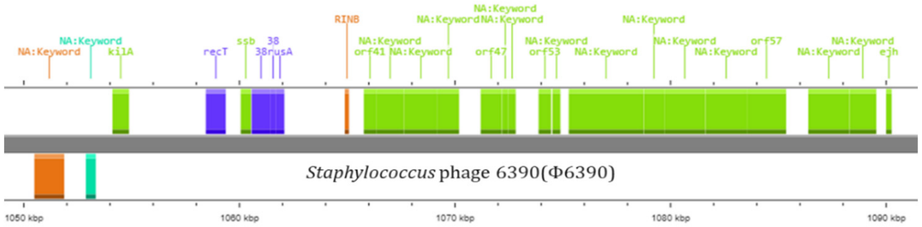

Figure S1 - *S. aureus* genomic comparison. (A) Multiple genome alignment comparing *S. aureus* TRS with RN6390 using Mauve [50] algorithm, red arrow pointing at genome inversion; (B) Prophage ϕ6390 features using Proksee [51].

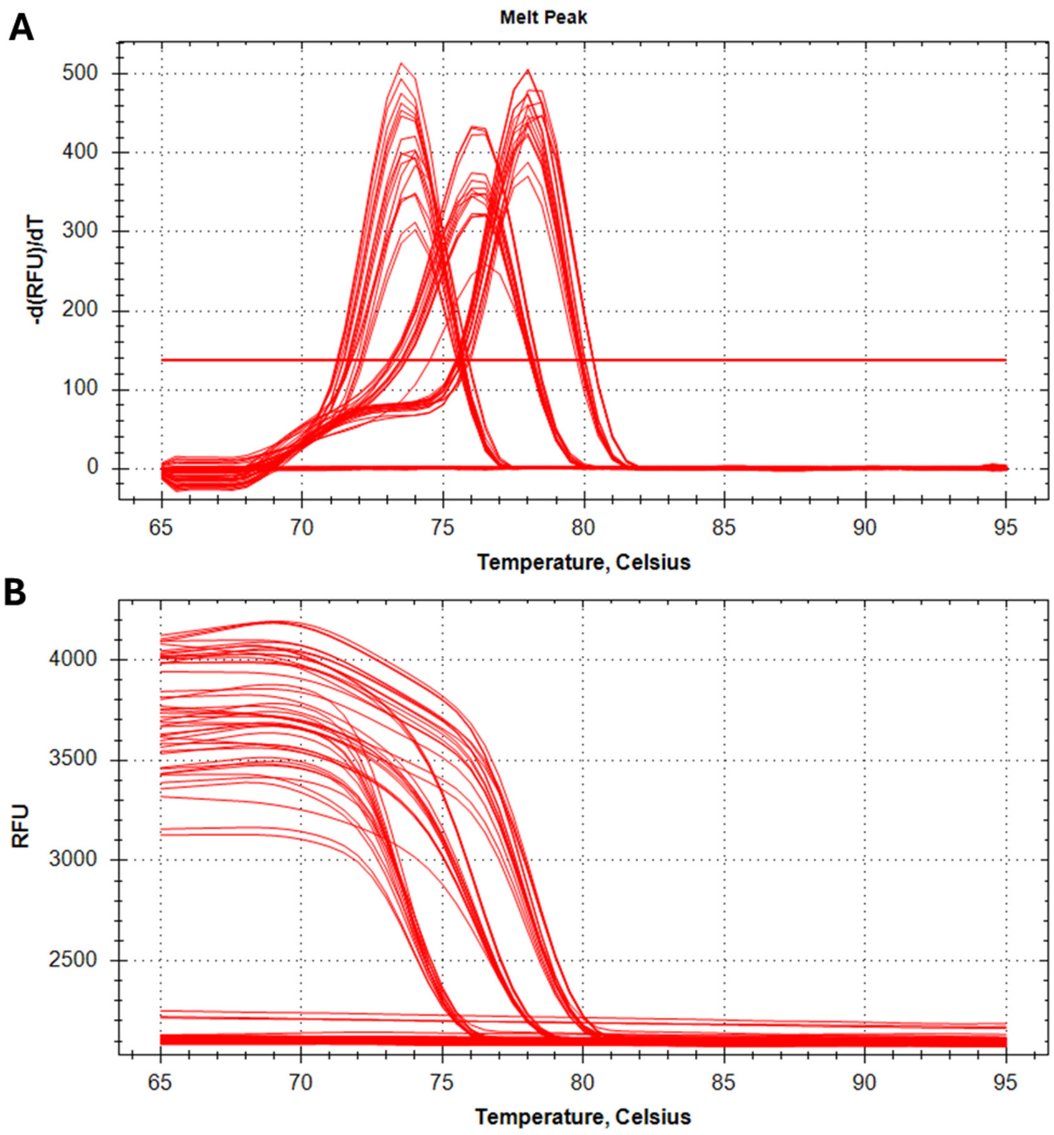

Figure S2 – *norB* primers efficiency for the TRS (CBS2016-06; CI/BAC/25/13/W; PS/BAC/169/17/W and PS/BAC/317/16/W). The graphs **A** and **B** demonstrate the reliability of the assay, indicated by the single-peak melt peaks and no peak corresponding to primer dimers or nonspecific products for the target gene or control in the melt curves, respectively.

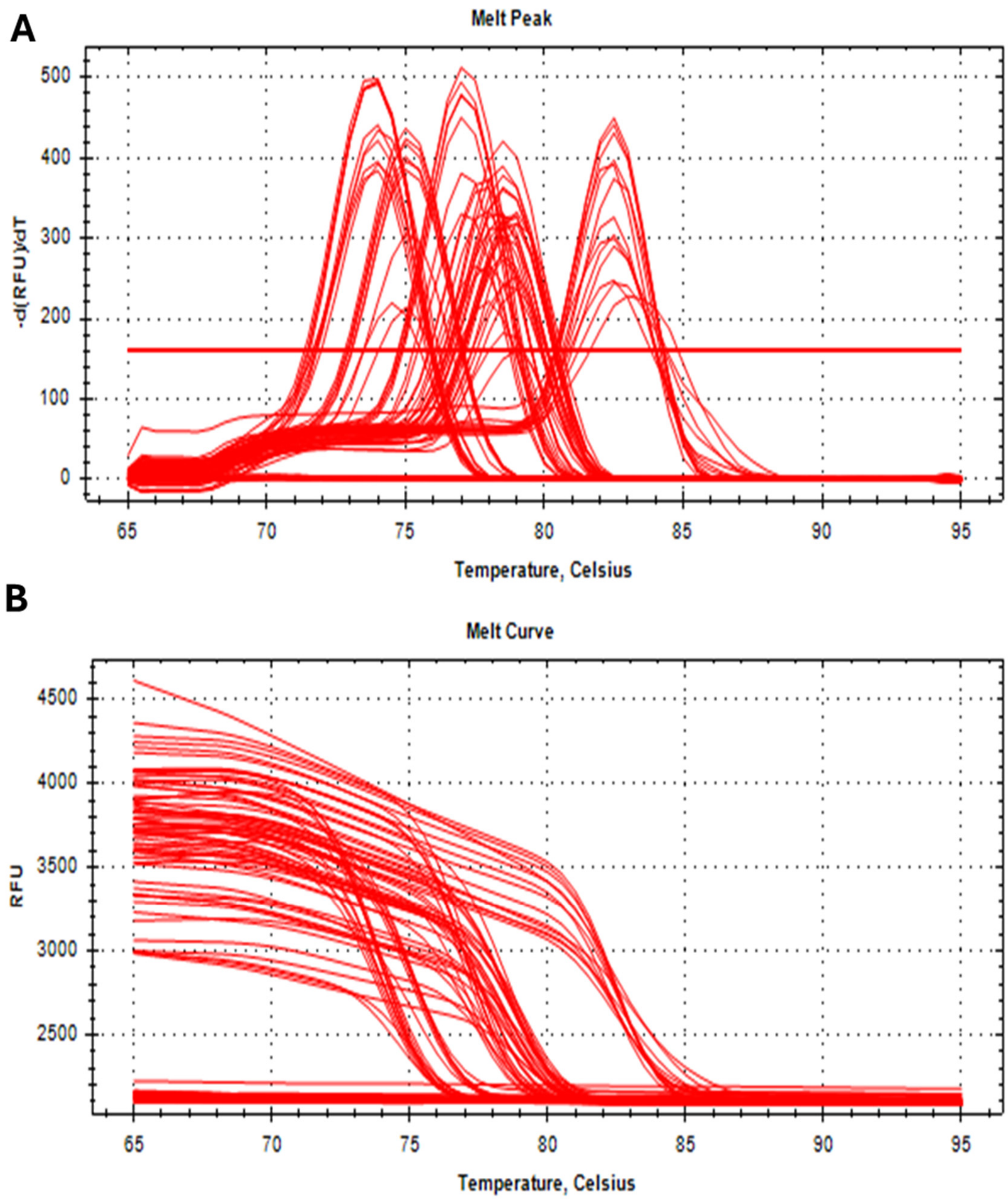

Figure S3 – Primer efficiency for RN6390 "wild type", and deletion mutants RN6390Δ*norB* and RN6390Δ*mgrA*. Primers sets (forward and reverse) 16S RNA, *norA*, *norB* and *norC*. The graphs **A** and **B** demonstrate the reliability of the assay, indicated by the single-peak melt peaks and no peak corresponding to primer dimers or nonspecific products for the target gene or control in the melt curves, respectively.
